# The role of the t-SNARE SNAP-25 in action potential-dependent calcium signaling and expression in GABAergic and glutamatergic neurons

**DOI:** 10.1186/1471-2202-9-105

**Published:** 2008-10-29

**Authors:** Lawrence CR Tafoya, C William Shuttleworth, Yuchio Yanagawa, Kunihiko Obata, Michael C Wilson

**Affiliations:** 1 Department of Neurosciences, University of New Mexico Health Sciences Center, Albuquerque, USA; 2 Department of Genetic and Behavioral Neuroscience, Gunma University Graduate School of Medicine, Maebashi, Japan; 3 Neural Circuit Mechanisms Research Group, Obata Research Group, RIKEN Brain Science Institute, Wako, Japan

## Abstract

**Background:**

The soluble N-ethylmaleimide-sensitive factor attachment protein receptor (SNARE) complex, comprised of SNAP-25, syntaxin 1A, and VAMP-2, has been shown to be responsible for action potential (AP)-dependent, calcium-triggered release of several neurotransmitters. However, this basic fusogenic protein complex may be further specialized to suit the requirements for different neurotransmitter systems, as exemplified by neurons and neuroendocrine cells. In this study, we investigate the effects of SNAP-25 ablation on spontaneous neuronal activity and the expression of functionally distinct isoforms of this t-SNARE in GABAergic and glutamatergic neurons of the adult brain.

**Results:**

We found that neurons cultured from *Snap25 *homozygous null mutant (*Snap25*^-/-^) mice failed to develop synchronous network activity seen as spontaneous AP-dependent calcium oscillations and were unable to trigger glial transients following depolarization. Voltage-gated calcium channel (VGCC) mediated calcium transients evoked by depolarization, nevertheless, did not differ between soma of SNAP-25 deficient and control neurons. Furthermore, we observed that although the expression of SNAP-25 RNA transcripts varied among neuronal populations in adult brain, the relative ratio of the transcripts encoding alternatively spliced SNAP-25 variant isoforms was not different in GABAergic and glutamatergic neurons.

**Conclusion:**

We propose that the SNAP-25b isoform is predominantly expressed by both mature glutamatergic and GABAergic neurons and serves as a fundamental component of SNARE complex used for fast synaptic communication in excitatory and inhibitory circuits required for brain function. Moreover, SNAP-25 is required for neurons to establish AP-evoked synchronous network activity, as measured by calcium transients, whereas the loss of this t-SNARE does not affect voltage-dependent calcium entry.

## Background

Regulated neurotransmission at chemical synapses underlies neural communication and is likely to contribute to complex brain functions, such as synaptic plasticity and memory storage. Neurotransmitter release requires fusion of synaptic vesicles that is mediated by the neuronal SNARE complex at release sites of presynaptic terminals [for review, see ref. [1,2]]. This core heteromeric protein assembly, comprised of the t-SNAREs syntaxin 1, and SNAP-25 situated at the target or plasma membrane and the v-SNARE VAMP-2/synaptobrevin on secreting vesicles, is responsible for membrane fusion that underlies the Ca^2+^-triggered neuroexocytosis that is required for AP-dependent neurotransmission signaling point-to-point communication between neurons, as well as the regulated secretion from neuroendocrine cells. Evidence suggests, however, that this neural SNARE complex may not be required for constitutive synaptic activity in the absence of presynaptic depolarization, although deletion of SNARE protein genes does alter characteristics of spontaneous neurotransmitter release events detected by recordings of AP-independent miniature postsynaptic currents or "minis" (mPSCs). For example, the analysis of neurons and neuroendocrine cells of SNAP-25 null mutant mice, generated by homologous recombination-mediated disruption of this t-SNARE gene [3], has demonstrated the selective abrogation of evoked neurotransmission, leaving constitutive release of neurotransmitter in catecholaminergic [4], GABAergic [5], glutamatergic and cholinergic systems [3] intact, despite varying effects on the amplitude and frequency of these transmitter-specific release events.

In addition to a well-documented role in membrane fusion for neuroexocytosis and neurotransmitter release, the t-SNAREs SNAP-25 and syntaxin 1 also associate with voltage-gated calcium channels (VGCCs) where they are thought to modulate steady-state inactivation of channel opening thereby regulating calcium currents in response to membrane depolarization [see ref. [6] for review]. In particular, SNAP-25 has been shown to specifically interact with one isoform of the P/Q-type VGCC (rbA) to limit calcium currents mediated by this channel [7]. Acute interference of SNAP-25 expression has been reported to lead to increased depolarization-induced calcium transients in cultured neurons [8]. Increased intracellular calcium ([Ca^2+^]_i_) would be expected to increase the frequency and amplitude of mPSCs [for review, see ref. [9]], yet previous evidence has shown that genetic deletion or BoNT cleavage of SNAP-25 can result in decreased mPSC frequency [10-13]. This raises the question of whether SNAP-25 plays any role in managing calcium influx through VGCCs.

Synaptogenesis and maturation of functional synaptic connectivity is accompanied by dramatically increased levels of SNAP-25 [14], as well as a significant change in the relative expression of transcripts encoding two isoforms of the protein that are produced by alternative splicing between divergent, tandem arranged copies of a single exon [15]. Interestingly, the representation of these two SNAP-25 isoforms differs markedly between neurons of the mature nervous system and in neurosecretory cells [15-21], suggesting that these isoforms are likely to impart physiological distinctions to presynaptic function that are ultimately required for the distinct properties of neurons that make up diverse components of neural circuitry. Consistent with this idea, the isoforms have been reported to promote differences in the recruitment of primed vesicles for neuroexocytosis in both chromaffin cells and hippocampal neurons [4,11], and in hippocampal short-term plasticity [22]. Nevertheless, whether such fine-tuning due to regulation of the expression of SNAP-25 and its isoforms plays such a role in sculpting properties of synaptic transmission in specific neuronal cell-types, and in particular excitatory glutamatergic or inhibitory GABAergic neurons, has not been fully resolved [5,8,23].

To explore this idea further, we investigated the role of SNAP-25 in synaptic communication and on calcium dynamics in neuronal cultures prepared from either *Snap25*^-/- ^mutants or control mice. We then extended our analysis to examine differences in the expression of SNAP-25 isoforms between glutamatergic and GABAergic neurons during development and within different functional networks. Our results support the idea that regulation of SNAP-25 contributes to the developmental fine-tuning of a universal SNARE complex required for mature, stimulus-evoked synaptic transmission both in cultured neurons and by major populations of excitatory and inhibitory neurons.

## Results

### Ca^2+ ^signaling in control and SNAP-25 deficient neurons

**Figure 1:**
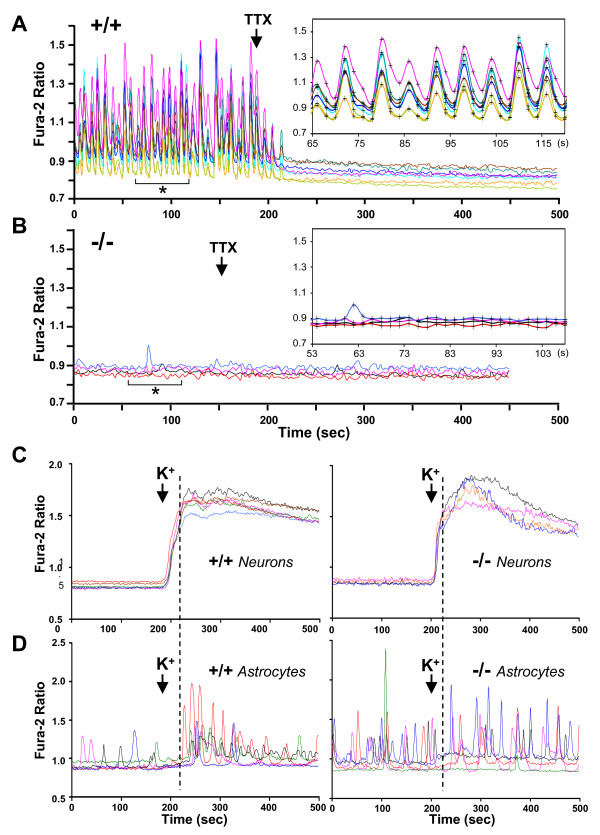
**Ca^2+ ^dynamics in hippocampal cell cultures**. Panel A, Spontaneous synchronized cytoplasmic Ca^2+ ^oscillations in *Snap25*^+/+ ^hippocampal neurons were abolished by addition of TTX (1 μM). Each trace is from a different neuron within the same culture dish. The inset shows a segment of the recording (prior to TTX, denoted by asterisk) at an expanded time base to show more clearly the synchrony of events. Panel B, Recordings from a culture derived from a homozygote *Snap25*^-/- ^fetus under identical conditions as illustrated in A. Spontaneous Ca^2+ ^transients in *Snap25*^-/- ^neurons were very rare and a single event in one neuron is shown at an expanded time base in the inset. Panel C, Depolarization with 55 mM K^+ ^(arrow) led to a sustained Ca^2+ ^elevation in neurons from both. *Snap25*^+/+ ^(left panel) and *Snap25*^-/- ^(right panel) cultures. TTX (1 μM) was included in both preparations, prior to K^+ ^application. Panel D, Astrocyte Ca^2+ ^oscillations from the same culture dishes illustrated in Panel C. Under control conditions, spontaneous events were observed in both *Snap25*^+/+ ^(left panel) and *Snap25*^-/- ^(right panel) cultures. Following K^+ ^application, an increase in frequency and amplitude of astrocyte events was observed in astrocytes from *Snap25*^+/+ ^but not in the *Snap25*^-/- ^preparation. A dashed line is drawn near the initial peak of neuronal Ca^2+ ^increases in the *Snap25*^+/+ ^preparation, to emphasize the relationship between neuronal and astrocyte signals. A similar relationship was not apparent in the *Snap25*^-/- ^culture. (See Fig. 3 for group astrocyte data).

Previous studies have established that cultured SNAP-25 deficient hippocampal neurons fail to trigger depolarization-dependent presynaptic vesicular endocytosis or to evoke postsynaptic currents [3,5,10]. To complement these investigations, we performed live-cell imaging of intracellular Ca^2+^dynamics in mixed cultures of hippocampal neurons and glial cells that were prepared from wild type *Snap25*^+/+^, heterozygous *Snap25*^+/-^, and homozygous *Snap25*^-/- ^fetuses. Other studies have shown that when cultured rodent CNS neurons adopt a synchronous pattern of synaptic network activity, it is reflected by oscillating Ca^2+ ^transients that can be monitored in the soma using the calcium indicator Fura 2 [24-27]. Ratiometric (350/380 nm) measurements were taken over neuronal and glial cell bodies (5–9 cells per coverslip) that were identified retrospectively by both morphology and a sustained Ca^2+ ^response to depolarization after bath application of media containing high K^+ ^(Fig. 1, and see Methods for details).

**Figure 2:**
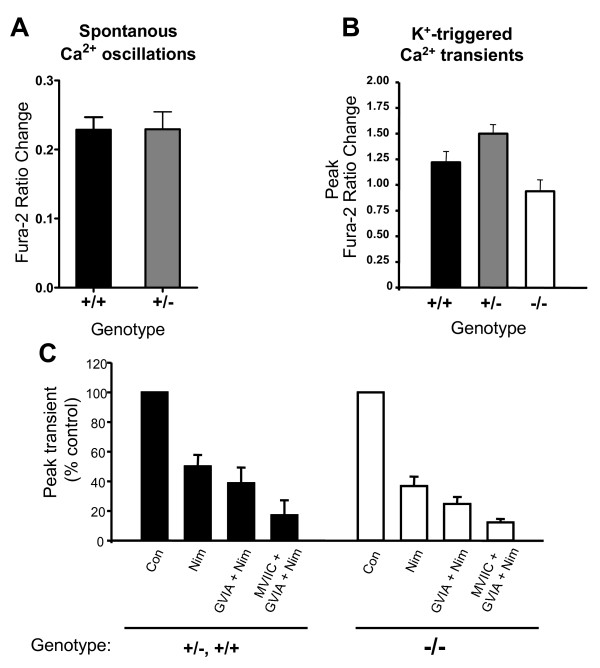
**Summary of neuronal Ca^2+ ^responses**. Panel A depicts summary data from 7 coverslips for each genotype, showing the mean amplitude of spontaneous Ca^2+ ^oscillations were not different between *Snap25*^+/+ ^(+/+) and *Snap25*^+/- ^(+/-) cultures. Panel B, amplitude of Ca^2+ ^elevations evoked by K^+^-depolarization in *Snap25*^+/+ ^(+/+), *Snap25*^+/- ^(+/-) and *Snap25*^-/- ^(-/-) cultures (n = 7,6,8, respectively). TTX (1 μM) was present throughout K^+ ^challenges. Panel C shows effects of sequential bath application of Ca^2+ ^channel blockers in 55 mM KCl containing media to assess the role of voltage-gated calcium channels in depolarization triggered Ca^2+ ^elevations of pooled results from *Snap25*^+/- ^and *Snap25*^+/+ ^(+/- and +/+) compared to *Snap25*^-/- ^(-/-) neurons. Responses were normalized to 55 mM K^+ ^challenges in control buffer. Con, control (TTX only), Nim, nimodipine (10 μM), conotoxins GVIA and MVIIC (1 μM).

Neurons of both wild type and heterozygous cultures exhibited characteristic synchronous oscillating Ca^2+ ^transients that had an overall frequency of 5.4 transients/min (range 1.4–8/min) (Fig. 1A). The amplitudes of oscillations produced by *Snap25*^+/+ ^and *Snap25*^+/- ^neurons were virtually identical (0.229 ± 0.018 versus 0.229 ± 0.025 ΔFura-2 ratio; n = 7 cultures of each genotype, 5 cells counted per culture, Fig. 2A), indicating that a reduced level of SNAP-25 in *Snap25*^+/- ^neurons did not affect the calcium currents measured from soma under these conditions. As expected, addition of tetrodotoxin (TTX, 1 μM) abolished neuronal Ca^2+ ^oscillations (Fig. 1A), demonstrating the dependence of these events on the propagation of action potentials and presumably synaptic release of neurotransmitters between cultured neurons. By comparison, as shown in Fig. 1B, cultures obtained from *Snap25*^-/- ^fetuses showed virtually no spontaneous Ca^2+ ^oscillatory activity (n = 8 cultures) in neurons. These SNAP-25 deficient neurons, however, did exhibit a robust Ca^2+ ^response after K^+ ^depolarization (Fig. 1C), consistent with the presence of functional VGCCs despite the lack of Ca^2+^-triggered evoked neurotransmitter release in the absence of neural SNARE proteins [3,28].

In *Snap25*^+/+ ^and *Snap25*^+/- ^cultures the resting [Ca^2+^]_i _level in neurons after the TTX block was significantly lower than that measured at the trough between oscillations prior to TTX exposure (Fura 2 ratio 0.861 ± 0.044, no treatment; 0.805 ± 0.031, TTX; n = 10 cultures, p < 0.008). However, in contrast to control cultures, TTX did not significantly affect resting [Ca^2+^]_i _in *Snap25*^-/- ^neurons (Fura-2 ratio 0.852 ± 0.023, no treatment, compared to 0.845 ± 0.021 with TTX; n = 7, p > 0.3).

To examine whether the mechanisms that underlie the generation of Ca^2+ ^transients were intact in the *Snap25*^-/- ^neurons, we compared their response with the Ca^2+ ^rise exhibited by control neurons after K^+^-induced depolarization. Bath application of 55 mM K^+ ^in the presence of TTX resulted in robust increases in [Ca^2+^]_i _levels in null mutant neurons that were not significantly different from control wild type or heterozygote neurons (Fig. 1C, quantified in Fig. 2B). Pre-incubation with selective voltage gated calcium channel blockers, moreover, did not show an overall effect on the relative contribution of L-, N- and P/Q-type channels as distinguished by the progressive addition of nimodipine (10 μM) and conotoxins GIVA and MVIIC (Fig. 2C). These results suggest that while SNAP-25 deficient neurons do not undergo Ca^2+ ^oscillatory behavior, presumably due to the absence of SNARE medicated evoked release, the general machinery required for Ca^2+ ^responses in the soma of these neurons has not been significantly altered.

### Astrocyte Ca^2+ ^dynamics

**Figure 3:**
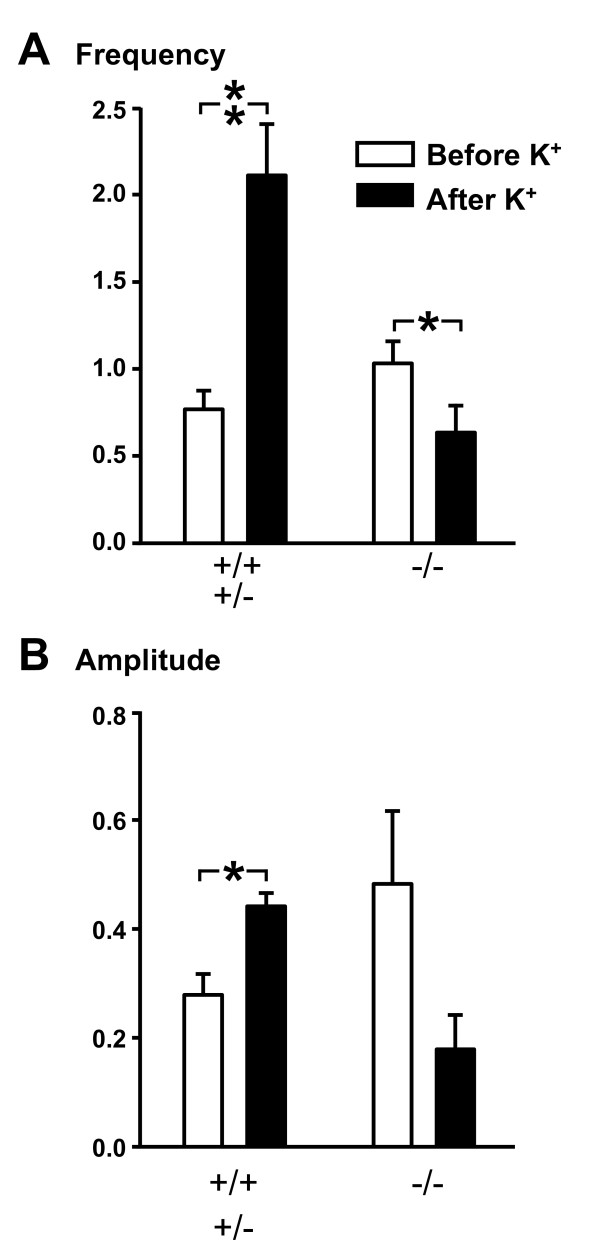
**Summary of astrocyte Ca^2+ ^oscillation responses**. Panel A, Frequency of Ca^2+ ^oscillations in astrocytes under control conditions (open bars) and during a 2 min time window following application of 55 mM K^+ ^(filled bars). Significant increases were observed in Snap25 wild type and heterozygous control cultures, in contrast to a decrease in the *Snap25*^-/- ^cultures. Panel B, The mean amplitude of individual astrocyte Ca^2+ ^oscillations was significantly increased following K^+ ^application in control cultures. Each bar represents mean ± SEM from 5 different cultures. Only astrocytes that displayed spontaneous events prior to K^+ ^were included in the analysis and responses from 3–5 cells per dish were averaged before calculating group means. (** P < 0.005; * P < 0.05).

We observed spontaneous Ca^2+ ^transients in astrocytes in the mixed cultures prepared from *Snap25*^+/+^, *Snap25*^+/- ^and *Snap25*^-/- ^mutant animals. As expected for astrocyte signals, Ca^2+ ^transients in these cells were not synchronized between individual astrocytes, and were not prevented by TTX. However, when high K^+ ^was applied to *Snap25*^+/+ ^and *Snap25*^+/- ^cultures in the presence of TTX, most preparations showed a clear increase in the frequency and amplitude of astrocyte Ca^2+ ^oscillations (Fig. 1D, grouped data shown in Fig. 3), that occurred in parallel with sustained Ca^2+ ^elevations in neurons. Astrocyte Ca^2+ ^signals were prominent in *Snap25*^-/- ^cultures and the characteristics of spontaneous events appeared similar to those observed in *Snap25*^+/+ ^and *Snap25*^+/- ^cultures, and were not prevented by TTX. However, in contrast to *Snap25*^+/+ ^and *Snap25*^+/- ^cultures, the average frequency of spontaneous astrocyte Ca^2+ ^oscillations significantly decreased (rather than increased) during high K^+ ^exposures (Fig 3A), although no significant change in the amplitude of the events was detected (Fig. 3B). This suggests that a lack of transmitter release from depolarized neurons impairs neuron-glia communication in these cultures.

### Developmental regulation of SNAP-25 in GABAergic neurons

While it has been demonstrated that SNAP-25 is expressed and required for stimulus-driven synaptic transmission by both glutamatergic and GABAergic neurons in fetal mouse brain and in culture [3,5,10,11], it has been also proposed that differential expression of this t-SNARE may lead to differences in calcium dynamics between inhibitory and excitatory synapses [8] and thereby possibly contribute to the physiological diversity observed between these neurons [for review, see [29]]. However, in the preceding experiments we did not detect a significant effect of SNAP-25 expression on the modulation of calcium responses in cultured neurons. Neuronal cultures do not exhibit the appropriate synaptic circuitry that is developed in the intact brain. We considered therefore whether the developmentally regulated isoforms of SNAP-25 [15,30] might be responsible, in part, for the distinctive synaptic properties of glutamatergic and GABAergic neurons. For example, while there is a general shift in the relative levels of the two isoforms during brain maturation, the expression of the earlier expressed SNAP-25a persists in most neuroendocrine cells, as well as in certain discrete neuronal populations [15-21]. Furthermore, the expression of the variant SNAP-25 isoforms has been shown to affect the size of the RRP in hippocampal neurons [11], as well as in adrenal chromaffin cells [4,31], and has been suggested to contribute to developmental changes in hippocampal short-term synaptic plasticity [22].

To examine whether two SNAP-25 isoforms are differentially expressed in GABAergic neurons and therefore might play a role in tailoring the distinct properties of synaptic activity in these neurons, we utilized *GAD67-GFP (Δneo) *transgenic mice that bear a knockin insertion of green fluorescent protein (eGFP) coding sequence at the glutamate decarboxylase 67 (GAD67) gene locus [32]. Expression of this fluorescent marker by virtually all GABAergic populations enabled us to distinguish and isolate GABAergic from GFP-negative, non-GABAergic and largely excitatory, neuronal populations. The level of expression of the isoforms was determined by real-time quantitative RT-PCR (qRT-PCR) using SNAP-25a and 25b transcript-specific primers [18]. Preliminary experiments established that the primer sets were equally efficient in amplifying the specific isoform sequences from cDNA templates, and RNA transcripts prepared from brains of SNAP-25a overexpressing mutant and control mice, as well as from cells transfected with cDNAs encoding the individual isoforms (see Methods), thereby validating the RT-PCR assay as a measure of the relative expression of the two isoform transcripts.

**Figure 4:**
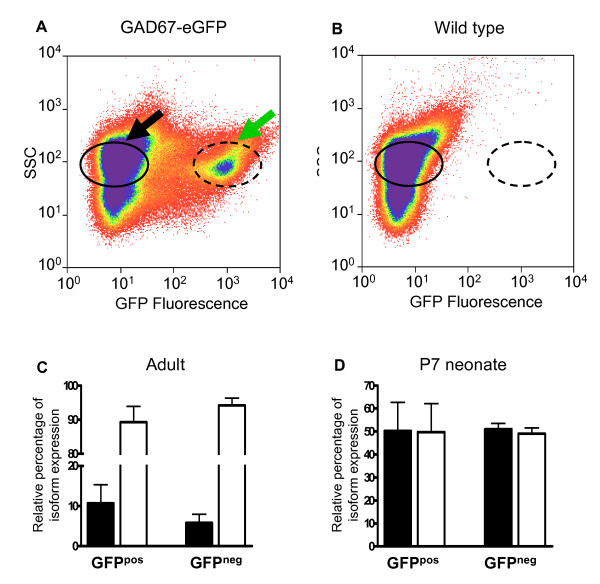
**Developmental regulation of SNAP-25 isoform expression in GABAergic neurons**. Panels A and B show a pair of graphs comparing cell populations prepared from acutely dissociated adult cortex of *GAD67-GFP (ΔNeo) *and nontransgenic wild type mice, respectively, after fluorescence-activated cell sorting (FACS). The selectivity of the fractionation is demonstrated by the distinct population of GFP-positive cells (dashed oval, panel A) that was readily distinguished in cells derived from *GAD67-GFP (ΔNeo) *mice, but was absent in preparations from wild type animals (panel B). The arrows in A indicate the two sorted cell populations, GFP-positive (green arrow) and -negative (black arrow), used for molecular analysis. Panel C shows the relative levels of SNAP-25a (solid bar) and SNAP-25b (white bar) RNA transcripts in adult neurons from GFP-positive (GFP^pos^, n = 4) and -negative (GFP^neg^, n = 2) populations. Panel D, SNAP-25 isoform RNA transcript expression in sorted immature neurons obtained from cortex of P7 neonates (GFP^pos^, n = 7; GFP^neg^, n = 4). Note that in contrast to the earlier developmental time point where comparable levels of the two isoform transcripts are expressed in both GFP^pos ^and GFP^neg ^cell populations, mature neurons of both populations express predominantly the SNAP-25b transcript.

A global description of isoform expression in the mature brain was first obtained by using fluorescence-activated cell sorting (FACS) to select eGFP-positive cells from freshly dissociated cerebral cortices of adult mice (Fig. 4). As expected, initial experiments demonstrated that a distinct GFP-positive (GFP_pos_) population of cells could be readily distinguished and isolated from GAD67-GFP (Δneo), but not control wild type littermates (compare Fig. 4 panels A and B). Subsequent qRT-PCR analyses were then carried out on the two fractions of sorted cells from transgenic animals to obtain GFP-negative (GFP_neg_, non-GABAergic cells) and GFP_pos _(GABAergic) populations (Fig. 4A, black and green arrow, respectively). The purity of the sorted cell populations was confirmed by qRT-PCR analysis for mRNA transcripts encoding the transmitter-specific transporters, VGLUT1 and VGAT. Our results, summarized in Table 1, demonstrate that a relatively high level of VGAT transcripts was detected in the absence of a VGLUT1 signal in GFP_pos _samples, and conversely, a similarly high level of expression of VGLUT1 was determined for GFP_neg _cells in which VGAT RNA was undetectable. Because the GFP_neg _cell population was not positively selected for any marker, it is likely composed of non-GABAergic neurons, the vast majority being glutamatergic, as well as astrocytes and other glial cell types. However, since SNAP-23, but not SNAP-25, is expressed in astrocytes [33,34] and little, if any SNAP-25 can be detected in oligodendrocytes [35], the amplification of SNAP-25 isoforms is primarily, if not exclusively, from glutamatergic neurons; a premise that is supported by the relatively high abundance of VGLUT1 transcripts detected by qRT-PCR.

**Table 1:** Relative percentage of VGLUT1 and VGAT mRNA levels compared to β-actin expression.

***Fluorescence-activated cell sorting***			
*Sample*	*GFP Phenotype*	*VGLUT1*	*VGAT*

Immature cortical cells	Negative	2.37%	n. d.*
	
	Positive	n. d.	7.89%

Adult cortical cells	Negative	3.91%	n. d.
	
	Positive	n. d.	9.51%

***Laser capture microdissection***			

*Region*	*GFP Phenotype*	*VGLUT1*	*VGAT*

Reticular Nucleus	Positive	n. d.	12.68%

Caudate	Positive	n. d.	14.07%

Purkinje cells	Positive	n. d.	15.39%

Hippocampal interneurons (CA1)	Positive	n. d.	12.45%

Hippocampal pyramidal cells (CA1)	Negative	11.95%	n. d.

Cerebellar granule cells	Negative	13.56%	n. d.

* n. d. (not detected) is considered less than 0.001% (i.e. Ct value > 10 cycles) of gene expression in comparable amounts of whole brain samples (see Materials and Methods).

As shown in Fig. 4C, qRT-PCR analysis of the GFP-expressing GABAergic cortical neurons obtained from adult mice showed nine-fold greater expression of SNAP-25b compared to SNAP-25a transcripts, indicating 90% of the total SNAP-25 mRNA population was composed of SNAP-25b and 10% of SNAP-25a coding mRNAs. Virtually identical results were obtained from the GFP_neg _cell fraction, consistent with previous findings based on an RNase protection assay of total adult brain RNA [15].

Since the relative expression of the SNAP-25 isoforms is dynamically regulated in cortex and other brain regions during development [15,16], we next investigated the relative abundance of the specific transcripts in GFP_pos _and GFP_neg _populations of cortical neurons prepared from P7 mice. As shown in Fig. 4D, in contrast to the greater abundance of SNAP-25b transcripts in adult brain, the level of the transcripts encoding the two isoforms was equivalent in both GABAergic and non-GABAergic populations of neonatal cortical cells. These results indicate GABAergic neurons in the developing and mature neocortex, principally represented by interneurons, express the same relative levels of SNAP-25 isoforms as the majority of cortical excitatory neurons that predominantly express SNAP-25b in the adult brain.

### Relative expression of SNAP-25 isoforms does not vary in different anatomical regions

**Figure 5:**
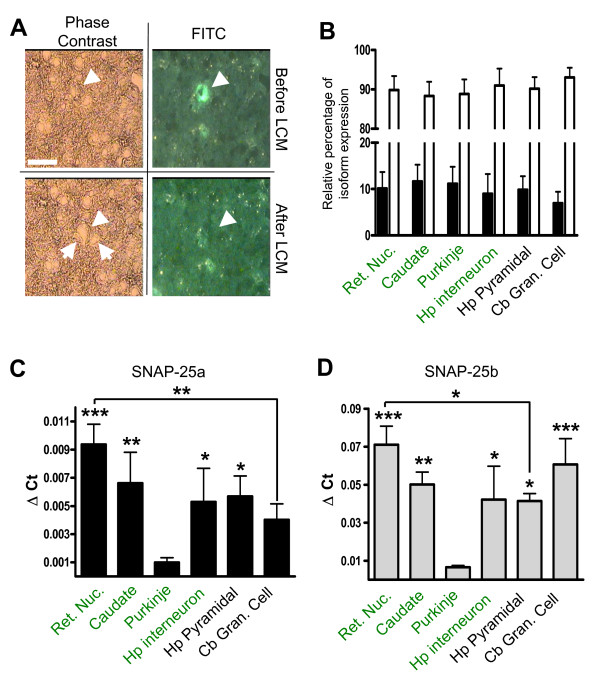
**SNAP-25 isoform expression varies between anatomical regions of the adult brain**. GFP-expressing cells from coronal sections of adult (P50) transgenic *GAD67-GFP (ΔNeo) *mouse brains were identified by epifluorescence and isolated by laser capture microdissection (LCM) from the reticular nucleus of the thalamus (Ret. Nuc.), caudate, cerebellum (Purkinje cells), and CA1 region of the hippocampus (Hp interneuron). In addition non-GFP expressing glutamatergic neurons collected from the pyramidal layer of the hippocampus (Hp Pyramidal) and granule layer of cerebellum (Cb Gran. Cell). The total RNA extracted from microdissected samples was assayed by real-time qRT-PCR analysis to assess transcript levels relative to β-actin as a control gene, as described in the Methods. Panel A is a representative micrograph of a GFP-expressing cell (arrowhead and hashed outline) before and after LCM, visualized with both phase contrast (left) and a FITC filter set (right). Note how neighboring cells (arrows) and tissue remain intact during the procedure. B, Histogram shows the relative ratio of SNAP-25a (solid bar) and 25b (white bar) RNA expression in selected brain regions (n = 6 pools taken from individual brains). Note that although, in all cases, SNAP-25b expression is significantly higher than SNAP-25a (***P < 0.001, Student's t-test), the ratio of isoform expression between anatomical regions does not change (n. s.; One-way ANOVA with Tukey post hoc comparison). Throughout the areas surveyed, however, the overall mRNA expression of SNAP-25a and SNAP-25b did vary widely (shown in Panels C and D, respectively). Statistical analysis with a one-way ANOVA and Tukey post hoc comparisons of total SNAP-25a and SNAP-25b transcript expression was carried out for each anatomical region (n = 3, except for cerebellar granule cells, n = 6). Asterisks denote significance of the difference between the level of SNAP-25 transcripts of each region compared to cerebellar Purkinje cells (* = p < 0.05; ** = p < 0.01; *** = p < 0.001); additional significant differences in isoform transcripts between GABAergic neurons of the reticular nucleus (Ret. Nuc.) and non-GABAergic neurons of the hippocampus (Hp Pyramidal) and cerebellum (Cb Gran. Cell) are also indicated.

The previous experiment examined the regulation of SNAP-25 isoforms within a fraction of cortical cells based globally on neurotransmitter phenotype. However, this analysis did not address whether the differential expression of the isoforms is common between different populations of GABAergic neurons. Therefore in order to evaluate the expression of the two isoforms within specific GABAergic and glutamatergic neuronal populations, we used laser capture microdissection (LCM) to isolate small groups of GABAergic and glutamatergic neurons from functionally distinct areas within the adult brain of *GAD67-GFP (Δneo) *transgenic mice. Using a fixation procedure optimized for LCM/qRT-PCR analysis (see Methods), eGFP-expressing cells in several representative anatomical regions were readily identified and single-cell microdissection allowed capture of these selected neurons without apparent excision of neighboring cells (Fig. 5A). To obtain a broad sampling of GABAergic populations, we collected pools of approximately 50 cells including projection neurons from the reticular nucleus of the thalamus, caudate, cerebellar Purkinje cells, as well as hippocampal interneurons located in both the stratum oriens and radiata of the CA1 region. In addition, we harvested non-GFP expressing glutamatergic neurons, specifically CA1 hippocampal pyramidal neurons and cerebellar granule cells, based on their distinct cellular morphology and location in well-defined areas of the brain. As before, we confirmed the purity of the selected GABAergic and glutamatergic neuron samples using qRT-PCR analysis of VGLUT1/VGAT expression (Table 1).

Analysis of cellular transcripts by qRT-PCR revealed no significant difference in the relative levels of SNAP-25a and SNAP-25b transcripts between GABAergic projection neurons and interneurons, regardless of anatomical location, and nonGABAergic (glutaminergic) hippocampal pyramidal and cerebellar granule cells (p = 0.25, one-way ANOVA, Tukey's Multiple Comparison Test, Fig. 5B). Overall, the predominance of SNAP-25b transcripts amongst these adult brain regions selected by LCM (88–93% of the total) was consistent with the isoform transcript levels obtained for adult cortical cells isolated by FACS (Fig. 4C), extending these findings to neuronal populations in the hippocampus, cerebellum and the subcortical regions of the thalamus and basal ganglia.

To examine the level of SNAP-25 mRNA expression in the selected cell populations, we further compared the qRT-PCR amplification of SNAP-25 isoform transcripts to β-actin RNA, taken as reference housekeeping gene. Amplification of β-actin RNA transcripts, as determined by Ct values was not significantly different among the different cell types (one way ANOVA, p = 0.1125). However, as shown in Fig. 5, panels C and D, the expression level of both SNAP-25a and SNAP-25b transcripts did vary significantly between various brain regions (SNAP-25a, p < 0.001; SNAP-25b, p = 0.0002, one-way ANOVA). In particular, although the relative levels of the isoform transcripts were similar, the major SNAP-25b RNA transcript, accounting for ~90% of the total SNAP-25 RNA, was significantly lower in GABAergic cerebellar Purkinje cells than other brain regions (Fig. 5D). Moreover, SNAP-25a transcripts in Purkinje cells were also greatly decreased relative to GABAergic neurons of the reticular nucleus and the caudate, as well as hippocampal interneurons and pyramidal neurons (Fig. 5C). Comparing the two major neuronal populations of the cerebellum, the abundance of SNAP-25b transcripts in Purkinje cells was more than an 8.5-fold lower, relative to β-actin, than in neighboring glutamatergic granule cells, despite virtually identical CT values obtained for β-actin RNA (Purkinje cell, 22.22 ± 1.26 versus granule cell, 21.77 ± 1.45). Between the neuronal populations surveyed, GABAergic neurons of the reticular nucleus of the thalamus exhibited a high level SNAP-25 isoform RNA expression with SNAP-25a being significantly increased compared with glutamatergic populations of cerebellar granule cells (p < 0.01) and SNAP-25b transcripts at greater abundance than hippocampal pyramidal neurons (p < 0.05), indicating that the absolute expression levels of SNAP-25 isoforms were also not tightly correlated with excitatory or inhibitory synaptic transmission. Taken together, these observations suggest that in mature neurons of the major excitatory glutamatergic and inhibitory GABAergic populations studied, the predominance of SNAP-25b-containing SNARE complexes neurons is a general, and possibly fundamental, characteristic, regardless of overall abundance of SNAP-25. Moreover, because the isoforms appear equally expressed by both glutamatergic and GABAergic neurons at P7 (Fig. 4D, and see ref. [15]), these data suggest that during development and maturation of neurocircuitry the regulation that drives the predominant expression of SNAP-25b occurs similarly in major populations of excitatory and inhibitory neurons.

## Discussion

### Developmental regulation leads to the predominant expression of SNAP-25b in adult glutamatergic and GABAergic neurons

The expression of SNAP-25 in adult rodent brain varies considerably between different neuronal cell groups [36] [reviewed in ref. [37]]. The cellular requirement responsible for the differential abundance of this basic component of the presynaptic exocytotic machinery, however, is less clear, although it likely results from the varied demands in the synaptic physiology of different neurons and their circuitry. For example, early studies based on qualitative *in situ *hybridization, suggested that while SNAP-25 was expressed robustly by cerebellar granule cells, SNAP-25 transcripts were undetectable in neighboring Purkinje cells [designated as MuBr8 in ref. [38]]. Using a more sensitive, quantitative qRT-PCR assay, we show here that the expression of SNAP-25a and 25b RNA transcripts compared to β-actin in Purkinje cells is indeed considerably lower than granule cells, and ranges from 6 to 10.5-fold less than the level detected in the other GABAergic neurons that were examined. Purkinje cells characteristically exhibit a pattern of tonic low frequency firing, accompanied by periodic high amplitude bursts [39]. Consequently, if SNAP-25 expression is driven by activity-dependent induction, the low abundance of SNAP-25 transcripts in Purkinje cells may reflect their intrinsic, relatively low synaptic activity. Nevertheless, our data only reflects mRNA levels in the soma, and not the abundance or activity of SNAP-25 within the presynaptic terminal, which may be also regulated by post-translational modifications, such as palmitoylation [40,41] or phosphorylation [42-45]. For example, in the hippocampus the abundance of SNAP-25 protein in presynaptic mossy fiber terminals of dentate gyrus granule neurons, which are highly active and contain a disproportionately large pool of releasable vesicles, appears much higher than in the terminal fields of neighboring pyramidal neurons [16,36], despite apparent lower levels of mRNA transcripts compared to CA3 pyramidal neurons. This suggests that trafficking, as well as functional modifications of this t-SNARE might play yet an additional critical role in the specialization of mechanisms that govern presynaptic neurotransmitter release.

While our results demonstrating the predominant expression of SNAP-25b isoform transcripts among neuronal populations of the adult mouse brain agree with a general shift in alternative splicing accompanying neuronal maturation [15], we were surprised to find no evidence for the differential expression of the two isoforms that has been observed previously between other regions of the CNS [15,16,19-21]. Among the brain regions we sampled, the prevalence of SNAP-25a transcripts was remarkably consistent (averaging 9.8% ± 1.7%, S.D.; see Fig. 5B) for neurons selected for either GABAergic or glutamatergic transmitter phenotype. In fact, cerebellar granule cells showed the most difference between these neuronal populations with SNAP-25a transcripts accumulating to only 7.0% of the total SNAP-25 RNA. One explanation for the discrepancy between our present results and the previous findings is that we selected neuronal populations primarily involved in fast, point-to-point neurotransmission, thus largely excluding neurons that primarily secrete other neurotransmitters and may depend more heavily on SNAP-25a expression [30]. For example, among the areas of the brain shown to exhibit preferential expression of SNAP-25a into maturity, the pituitary and hypothalamus are chiefly populated by neurosecretory neurons that are characterized by their release of hormones and other neuropeptides [20,46]. Neurons in these areas maybe more comparable to other neuroendocrine cells, such as adrenal gland and pancreatic beta cells, that persistently express high levels of SNAP-25a in the adult [17,18,47], suggesting that the preference for one isoform may reflect a mechanism that tailors exocytotic machinery to secretory properties. Consistent with this idea, expression of SNAP-25b leads to the greater recruitment of vesicles to the readily releasable pool in hippocampal neurons compared to SNAP-25a [11], and similarly stabilizes a larger pool of vesicles for catecholamine secretion in adrenal chromaffin cells [4]. Interestingly, the expression of the isoforms also appears to be responsive to synaptic activity. Depolarization of dentate gyrus granule cells has been shown to induce expression of SNAP-25b rather than SNAP-25a [48], whereas activation of neurosecretory magnocellular hypothalamic neurons has been reported to increase SNAP-25a expression exclusively [20]. Selection of a particular SNAP-25 isoform, therefore, may provide a functional advantage in refining the exocytotic machinery necessary for different modes of vesicular release.

Consistent with previous studies [15,16], we find a similar developmental profile of SNAP-25 isoforms in GABAergic (GFP_pos_) and nonGABAergic (GFP_neg_, glutamatergic) neurons in the cortex with equal representation of SNAP-25a and -25b transcripts in neonates leading to the predominant expression of SNAP-25b in the adult. Interestingly, SNAP-25b is also the predominant isoform expressed in dentate gyrus granule cells [16] that have been shown to simultaneously release both GABA and glutamate [49]. Several recent studies have reported, however, that although SNAP-25 was detected initially in interneurons of the developing hippocampus, the expression waned as these GABAergic neurons mature in culture and appeared to be undetectable at these synapses, as well as synapses of other GABAergic neurons in the adult brain [8,23,43] [for review, see ref. [37]]. Although the reason for discrepancy between these observations and our previous results demonstrating co-localization of SNAP-25 immunoreactivity with GABAergic markers in several GABAergic neuronal populations [5] remains to be resolved, our present findings indicate that SNAP-25b RNA transcripts are, in fact, robustly expressed by GABAergic neurons isolated from cortex, thalamus, caudate, as well as by hippocampal interneurons at a level comparable to that seen in excitatory, glutaminergic neurons. Taken together with other studies [5,10,11], these results provide additional evidence that SNAP-25b is a key component of the neural SNARE complex responsible for both GABAergic and glutamatergic transmission in mature neurons.

### Alterations of calcium dynamics in SNAP-25 deficient neuronal cultures

Consistent with the idea that evoked synaptic activity is required to establish network activity between cultured neurons, we found that spontaneous, synchronized calcium oscillations were absent in dispersed hippocampal cultures prepared from *Snap25*^-/- ^mice. These SNAP-25 deficient mutant neurons were, nevertheless, able to generate calcium transients after depolarization. Interestingly, the amplitude of spontaneous synchronous calcium spikes in cultures from heterozygous null mutants, expressing reduced levels of SNAP-25, did not differ substantially from wild type neurons. Moreover, the magnitude of the calcium response evoked in SNAP-25 null mutant neurons by exposure to high K^+ ^depolarizing media was also equivalent to the responses measured in control *Snap25*^+/- ^and *Snap25*^+/+ ^neurons. Previous studies have shown that SNAP-25 is associated with N and P/Q type voltage gated calcium channels [for review, see ref. [6]], and specifically impedes calcium currents through P/Q type channels activated in response to action potential-like stimuli [7]. In our experiments, however, we did not observe a significant enhancement of the relative contribution of MCVII toxin sensitive P/Q type channels to the overall calcium response in *Snap25*^-/- ^compared to control neurons. This suggests that the modulation of these calcium channels by SNARE protein interactions does not occur in the soma, but may be limited to presynaptic terminals, which lie beyond the level of resolution achieved in these experiments. In contrast to these results, Matteoli and colleagues have reported a *Snap25 *genotype-dependent difference in calcium responsivity with higher peak calcium responses evoked from hippocampal neurons prepared from homozygous *Snap25 *null mutants compared to wild type, and intermediate values from neurons heterozygous for the null mutation [43]. One possibility that could contribute to these different findings may be the variability seen in the viability of SNAP-25 deficient neurons in culture [see ref. [3,10,11]]. In an effort to control for this variability, we averaged the mean calcium peak response exhibited by 6–8 individual cultures (after assaying 5–10 neurons per field) for each genotype. Moreover, to control for differences in the complexity of neurite extension that is evident between cultures, and more importantly genotypes, we restricted our measurements to fura-2a responses imaged over cell bodies, thus avoiding the contribution of calcium transients in dendrites.

Astrocytes have been proposed to join with presynaptic terminals and postsynaptic spines to form a "tripartite synapse," that enables bidirectional communication between glia and neurons [for review, see ref. [50]]. Indeed, in most cultures of SNAP-25 expressing neurons, neuronal depolarization was accompanied by clear increases in astrocyte Ca^2+ ^oscillatory activity. This correlation could be due to a number of factors, including direct effects of K^+ ^triggering depolarization on astrocytes. Nevertheless, since astrocytes do not express SNAP-25, but utilize the independent t-SNARE homologue SNAP-23 [34], this deficit in astrocyte Ca^2+ ^responsiveness in SNAP-25 deficient cultures provides further evidence for the role of AP-dependent synaptic transmission in neuronal-glia communication.

## Conclusion

Overall, our results are consistent with the idea that SNAP-25b serves as the predominant t-SNARE responsible for action potential-dependent neurotransmission in the major excitatory glutamatergic and inhibitory GABAergic neurons in the mature brain. In addition, we conclude that while deficits in SNAP-25 do not selectively dysregulate specific voltage-gated calcium channels at the soma, this neural SNARE component is needed to maintain normal synaptic activity that is reflected by calcium signaling between neurons and within a neural-glial network.

## Methods

### Animal procedures

Heterozygote *Snap25 *null mutant mice (JAX strain designation B6.129X1-*Snap25*^*tm*1*Mcw*^/J; [ref. [3]]) have been maintained by brother:sister mating after 7 backcross generations to C57Bl/6 at the UNM HSC Animal Resource Facility. To prepare neuronal cell cultures, *Snap25 *homozygote null mutants (*Snap25*^-/-^), heterozygote *Snap25*^+/- ^and wild type (*Snap25*^+/+^) fetuses were collected from timed pregnant dams of heterozygote matings. At E17-E18 (plug date, day 0) pregnant animals were killed by rapid cervical dislocation and decapitation as described previously [3,5]. Fetuses were removed sequentially from the uterus, and *Snap25*^-/- ^fetuses were initially identified by the absence of a response to a pinch to the hindlimb. PCR genotyping [3] was used to confirm null *Snap25*^-/- ^mutants, and to distinguish between heterozygote *Snap25*^+/- ^and homozygote *Snap25*^+/+ ^fetuses that served as control littermates. Pups were quickly decapitated and their brains were removed and placed in ice-cold PBS. For FACS analysis and laser capture microscopy studies (see below for Methods), mice were euthanized with phenobarbital. All procedures were performed in accordance with guidelines of the University of New Mexico Health Sciences Center Laboratory Animal Care and Use Committee, and the National Institutes of Health.

### Imaging of intracellular Ca^2+ ^transients

Hippocampal neurons were isolated from individual E17.5 fetal mice and grown as dispersed mixed cell cultures plated on poly-L-lysine/laminin-coated 12 mm coverslips (four coverslips per animal; 50,000 cells/coverslip) for 9–11 days (9–11 DIV) as described previously [3]. Cytosolic Ca^2+ ^levels were assessed using the high-affinity ratiometric indicator Fura-2. Cultures were loaded at room temperature with 3 μM Fura-AM for 20 min in HEPES buffer (130 mM NaCl, 5 mM KCl, 2 mM CaCl_2_, 1 mM MgCl_2_, 11 mM Glucose, 10 mM HEPES pH 7.6) and then rinsed for 20 min in HEPES to allow for deesterification of indicator. Cultures were then transferred to the recording chamber and superfused with HEPES at 2 ml/min at room temperature. Cultured were allowed to equilibrate to the recording conditions for 20 min before recording was begun. Depolarization-induced Ca^2+ ^increases were evoked by rapid complete exchange of the chamber contents with 55 mM K^+ ^solution. 10 min intervals in normal HEPES buffer were maintained between repetitive challenges. In the absence of any inhibitors, this procedure produced reproducible Ca^2+ ^responses throughout the time course of these experiments. Antagonists were applied to cultures 5 min before the onset of K^+ ^challenges, and maintained in the recording solutions thereafter. Fura-2 excitation was achieved using 350/380 nm pairs (40 ms each) delivered from a monochromator (TiLL Photonics GmbH, Grafeling, Germany) via a 40 × WI objective (Olympus, N.A. 0.8). Fluorescence emission (510 nm) was detected using an interline transfer cooled CCD (TiLL Imago). Image pairs were background-subtracted and then ratioed (Till Vision v 4.0).

### Separation of GAD67-GFP (ΔNeo) cells by Fluorescence-activated Cell Sorting

Cerebral cortices were washed twice in PBS and then incubated in a solution containing papain (2 mg/ml; Sigma, St. Louis, MO) and Hibernate A media (without CaCl_2_; BrainBits LLC, Springfield, IL) for 30 minutes at 30°C. Digested tissue was then transferred to Hibernate A alone followed by mild trituration through both wide-bore and fine-tipped pipettes. Prior to flow cytometry, the cells were filtered and resuspended in 5 ml of ice-cold PBS.

Flow cytometry was performed using the MoFlo High-Performance Cell Sorter (Dako Inc., Fort Collins, CO) equipped with a 488 nm excitation laser and a 530–540 nm band pass filter. eGFP expressing cells were sorted at a rate of 1000 events/sec through a 100 μm nozzle. Gating threshold parameters were selected was based on optimal measurements of side scatter (SSC) and GFP fluorescence. Two separate fractions, either GFP-positive or GFP-negative cells, were collected for each cortical sample.

The isolated cells were centrifuged at 500 × g for 5 min, supernatant was removed, and tissue pellets were homogenized in 4 M guanidinium thiocyanate (GTC), 50 mM Tris pH 8.5, 10 mM EDTA, 0.5% sarcosyl, and 200 mM β-mercaptoethanol. After mixing in 1/10 volume of 2 M sodium acetate (pH 4.0), RNA was extracted using 1/5 volume of a 24:1 chloroform/isoamyl alcohol mixture and 1.0 volume of acid phenol (pH 4.3). Samples remained on ice for 20 min, followed by centrifugation at 4°C for 15 minutes. The aqueous phase was collected and the RNA was precipitated by addition of isopropanol (1.0 volume). Samples were placed at -20°C for 1 hour, centrifuged, and washed with 70% ethanol before resuspension in DEPC-treated water. RNA samples were then stored at -80°C until use.

### Laser capture microdissection

While irreversible cross-linking fixatives, such as paraformaldehyde, provide excellent conservation of GFP fluorescence, throughout subsequent tissue processing, it greatly compromises RNA integrity. Therefore, as an alternative, we used the reversible cross-linking fixative, DSP to balance preservation of mRNA levels while retaining detectable GFP fluorescence in the tissue [51]. After euthanization with phenobarbital, mice were transcardially perfused with 0.1 M phosphate buffer (PB) to flush out brain vasculature, followed by a 1 mg/ml solution of dithiobis(succinimidyl)propionate (DSP; Pierce Biotechnology, Rockford IL) in 0.1 M PB. To avoid precipitation of DSP, a 10× stock solution made in DMSO was added slowly to 0.1 M PB, and filtered just before use. After perfusion, the brains were removed and placed in DSP/0.1 M PB solution overnight at 4°C for postfixation. Brains were cryoprotected by immersion in 30% sucrose for 18–24 hours at 4°C, and embedded in Tissue Tek OCT compound (Sakura Finetek, Torrance, CA). 10 μm coronal sections were cut using a Microm HM 550 cryostat set at -30°C (Richard-Allan Scientific, Kalamazoo, MI), mounted on uncoated glass slides, and stored at -80°C until use.

For laser capture microdissection, neurons from transgenic *GAD67-GFP (ΔNeo) *mice were harvested using a Pixcell II apparatus (Molecular Devices, Sunnyvale, CA) connected to a Nikon microscope using a 40× objective (N. A., 0.6) and a FITC filter set. Before microdissection, the sections were dehydrated by immersion through 70%, 95%, and 100% ethanol (30 sec each), followed by xylene (5 min), and final air-drying (10 min). GABAergic cells from different brain regions were identified by epifluorescence, and pyramidal neurons of the hippocampal CA1 region and granule cells from the cerebellum were identified by their distinctive cellular morphology using phase contrast optics. Pools of approximately 50 individually dissected cells from each anatomical region were captured on a single CapSure^® ^HS LCM Cap (Molecular Devices) using multiple pulses at a laser power setting of 90 mW, a spot size of 7.5 μm, and duration of 0.1 msec. Each pool of cells, collected from a single animal, was considered as a single, individual sample. To isolate total RNA extracts free from genomic contamination, we used reagents and protocols of the PicoPure RNA isolation kit (Molecular Devices) and the RNase-free DNase kit (Qiagen, Valencia, CA).

### Complementary DNA synthesis and quantitative PCR assay

Complimentary DNA (cDNA) was synthesized using 25 pmol oligo(dT)_12–18 _as a primer (USB, Cleveland, OH) and Moloney murine leukemia virus (MMLV-I; USB) reverse transcriptase using reagents provided by the manufacturer. Briefly, the entire RNA sample was incubated with the primer at 75°C for 5 min, cooled on ice and added to a reaction buffer containing 100 units of reverse transcriptase, M-MLV Reaction Buffer (diluted to 1×, supplied by USB; final concentration containing 50 mM Tris-HCl, pH 8.3, 79 mM KCl, 3 mM MgCl_2_, 10 mM DTT), and 0.5 mM dNTPs in a volume of 25 μl. The samples at 42°C for 30 min, followed by heat inactivation of the reverse transcriptase at 95°C for 5 min.

Quantitative real-time PCR (qRT-PCR) was carried out on cDNA samples using SYBR green master mix (SuperArray, Frederick, MD). The primer set for VGAT was obtained from SuperArray. Primer sets for VGLUT1, β-actin, and those specific for SNAP-25 isoform transcripts (using a pan SNAP-25 forward with either SNAP-25a [18] or SNAP-25b reverse primers), shown in Table 2, were designed or evaluated using software and services from Integrated DNA Technologies (Coralville, IA). Real time quantitative PCR was carried out in a ABI 7000 Sequence Detection System real-time PCR thermocycler (Applied Biosystems, Foster City, CA, USA), under the following cycling parameters: 50°C for 2 min, followed by 95°C for 10 min, then 45 cycles (95°C for 15 sec; 64°C for 45 sec) preceding a dissociation curve. All reactions were performed in triplicate, and each experiment was independently repeated a minimum of three times.

**Table 2:** Sequence of oligonucleotides

*Primer*	*Bases*	*Sequence (5'-3')*	*Reference*
VGLUT1 forward	747–776	AGGAGGAGCGCAAATACATTGAGGATGCCA	BC054462
VGLUT1 reverse	866-837	TGATGGCATAGACGGGCATGGACGTAAAGA	

β-actin forward	1033–1062	TGCTCTGGCTCCTAGCACCATGAAGATCAA	NM007393
β-actin reverse	1231-1202	AAACGCAGCTCAGTAACAGTCCGCCTAGAA	

Pan SNAP-25 forward	58–82	CAGCTGGCTGATGAGTCCCTGGAAA	AB003991
SNAP-25a reverse	207-172	TTGGTTGATATGGTTCATGCCTTCTTCGACACGATC	AB003992
SNAP-25b reverse	267-234	CACACAAAGCCCGCAGAATTTTCCTAGGTCCGTC	

**Figure 6:**
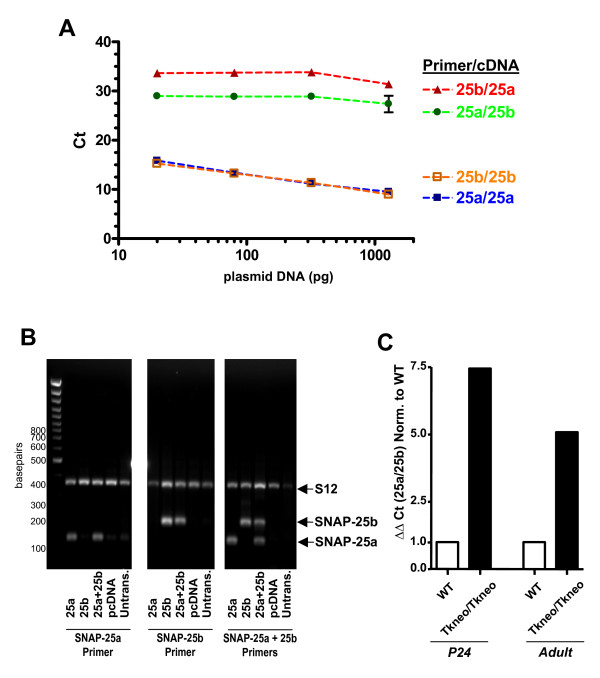
**Quantitative real-time PCR assay of SNAP-25 isoform transcripts**. Quantitative RT-PCR demonstrated specific and equivalent amplification of SNAP-25 isoform sequences using primers designed to exploit nucleotide sequence differences between SNAP-25a and 25b (see Table 2). Panel A, Calibration curve of quantitative RT-PCR performed using primers sets on SNAP-25 isoform cDNA. Ct values (triplicates ± SEM) obtained for each primer/cDNA plasmid pair were plotted versus the amount of DNA template on a log scale to demonstrate the linear relationship between amplification and DNA input. Robust and equal amplification of each SNAP-25 isoform cDNA was detected only with the appropriate primer set and corresponding plasmid (SNAP-25a: 25a/25a, blue closed squares; and SNAP-25b 25b/25b, orange open squares). Only non-specific, negligible amplification (CT values >30) was obtained from non-corresponding primer/plasmid sets (e.g. 25a/25b, green triangles; 25b/25a red circles). Panel B, RT-PCR performed on RNA of transfected cells. COS7 cells (1.5 × 10^5 ^cells/well of a 12 well plate) were transfected with equivalent molar amounts (~1 μg/well) pCDNA3 expression plasmids bearing SNAP-25 isoforms [15], or the empty vector (pCDNA), using Lipofectamine (Invitrogen, Carlsbad CA, USA). cDNA prepared from RNA (1 μg) extracted from the transfected or untransfected (Untrans) control cells was amplified using the indicated isoform specific reverse primer, either individually or together (25a+25b), by conventional end-point reverse transcriptase PCR (40 cycles, see Methods). Amplification using a primer set to S12 rRNA protein transcripts served as a positive control. The size of the isoform specific amplicons (SNAP-25a 149 bp; SNAP-25b, 176 bp) is due to the different positions of isoform-specific reverse primers relative to the common forward primer, panSNAP25. The lack of a band in the mismatched primer/template lanes reflects the specificity of the PCR reaction. Panel C, Quantitative RT-PCR using total RNA preparations from cortex of wild type (WT, white bar) and SNAP-25a overexpressing knockin mutant (*Snap25*^tkneo^, black bar) mice. The relative overexpression of SNAP-25a transcripts in the homozygous mutant (Tkneo/Tkneo) samples, expressed as ΔΔCt (25a/25b) normalized to wild type (WT), was calculated from the SNAP-25 isoform amplification relative to β-actin (ΔCt value) and then deriving the relative ratio of their amplification rates (ΔΔCt). The ratio of SNAP-25a to SNAP-25b (e.g. 25a/25b) obtained from the mutant was then normalized to wild type (set at 1.0) to obtain a fold increase of SNAP-25a expression in these mutant mice. This increased level of SNAP-25a isoform transcripts in the mutant cortex RNAs, seen in both young (P24) and adult (P124), is consistent with values previously reported for these mice [22] confirming the specificity of each primer set and their use in assaying the expression of SNAP-25 isoforms in brain tissue.

The SNAP-25 isoform primer sets were first evaluated by performing quantitative real-time PCR to generate a standard curve on plasmid DNAs containing cDNAs for the entire open-reading frame of each isoform mRNA (Fig. 6A), demonstrating that the isoform primers were both specific and amplified SNAP-25 isoform sequences at equivalent rates. Similarly, the isoform-specific primers were tested and found to specifically amplify cDNA prepared from COS 7 cells transfected with plasmids encoding either SNAP-25a or 25b transcripts (Fig. 6B). To assess the ability to quantify SNAP-25 isoforms from brain tissue, we also compared amplification from cDNAs prepared from cortex of *Snap25*^+/+ ^and a *Snap25 *knockin mutant (*Snap25*^tkneo/tkneo^) that overexpresses SNAP-25a transcripts [22]. As shown in Fig. 6C, qRT-RCR was readily able to distinguish a 7.5-fold and 5-fold greater expression of SNAP-25a transcripts in young P24 (post-weaning) and adult (P124) knockin mice, respectively, relative to wild type, which is comparable to level of overexpression of SNAP-25a transcripts previously determined for these mice based on isoform-specific cleavage by restriction endonucleases of total SNAP-25 PCR amplified cDNA [see ref. [22]]. Taken together, these results demonstrated that the RT-PCR assay with these primers provided a quantitative measure of the relative expression of transcripts encoding the two SNAP-25 isoforms.

Relative transcript levels were calculated from data within the linear range of cDNA amplification, as determined automatically by the instrument software. Within each sample, a 2^ΔCt ^or 2^ΔΔCt ^analysis method was used to compare the expression levels of target genes after normalizing to amplification of β-actin transcripts as a housekeeping gene. In the series of experiments evaluating SNAP-25 isoform expression in cells harvested from different brain regions by LCM (Fig. 5), the Ct values for β-actin were not significant between cell types (n = 6 samples of each cell type, one-way ANOVA, p = 0.1125), indicating that the β-actin levels could be taken as a reference to normalize SNAP-25 transcript levels between these brain regions. For each sample, the absence of amplification from genomic DNA was confirmed by omitting reverse transcriptase during cDNA synthesis before qRT-PCR. Background signal in negative control samples was defined as not detectable based either by failing to cross the detection threshold automatically set by software parameters, or if the amplification was >10 cycles beyond the Ct value of signal found in experimental or positive control samples. Data was analyzed using Prism 4.03 (GraphPad Software) as group means with a Student's t-test or one-way ANOVA.

## Authors' contributions

LCRT prepared neuronal cultures, designed and carried out the molecular analysis of GABAergic and glutamatergic neurons, and drafted the manuscript. CWS performed the calcium imaging experiments. YY and KO provided the GAD67-eGFP mice. MCW was responsible for the overall design of the study, participated in the calcium imaging and helped to draft the manuscript. All authors read and approved the final manuscript.
